# 3,4-Dihydroxyphenylethanol ameliorates lipopolysaccharide-induced septic cardiac injury in a murine model

**DOI:** 10.1515/biol-2021-0125

**Published:** 2021-12-20

**Authors:** Lu Zhang, Kun Wen, Zhiqiang Zhang, Chengen Ma, Ni Zheng

**Affiliations:** Department of Intensive Care Unit, The Second Hospital of Shandong University, Jinan, Shandong Province, 250033, China; Department of Clinical Laboratory, Shandong Provincial Hospital Affiliated to Shandong First Medical University, No. 324 Jingwu Weiqi Road, Jinan, Shandong Province, 250021, China

**Keywords:** DOPET, sepsis, LPS, cardiac damage, inflammation, apoptosis

## Abstract

3,4-Dihydroxyphenylethanol (DOPET) is a polyphenol found in olive oil. The present study evaluated the protective role of DOPET on LPS provoked septic cardiac injury in a murine model. Four groups were used in the study (*n* = 3): control, LPS, DOPET alone, and DOPET + LPS. LPS (15 mg/kg; i.p.); they were used to induce cardiac sepsis. The cardiac markers like LDH, CK-MB, and troponin-T, as well as inflammatory cytokines like TNF-α and IL-6 were measured in the serum. The antioxidants and oxidative stress parameters were measured in cardiac tissues. RT-PCR and western blot methods were done to evaluate the expression of inflammatory mediators and apoptotic markers. DOPET significantly decreased the cardiac markers (LDH, CK-MB, and troponin-T) and TNF-α and IL-6 level in the serum. DOPET effectively reduced the levels of MDA and NO in LPS intoxicated rats. DOPET also increased the levels of antioxidants like SOD, CAT, GPx, and GSH in LPS intoxicated rats. The mRNA levels of TNF-α, IL-6, and NF-κB were significantly downregulated by DOPET in cardiac tissues of LPS rats. The protein expression of Bcl-2 was upregulated, and Bax and caspase-3 were downregulated by DOPET. DOPET effectively attenuates LPS-induced cardiac dysfunction through its antioxidant, anti-inflammatory, and anti-apoptotic mechanisms.

## 1 Introduction

Sepsis is a noxious systemic inflammatory, infectious disease mediated by the presence of microorganisms that cause high hospital-related mortality [1]. The mortality observed during sepsis is due to multiple organ failure and has a rate between 12 and 40% [2]. Sepsis is broadly divided into three stages based on the severity of symptoms. It includes sepsis, severe sepsis, and septic shock. Among all, septic shock is the most complicated and common cause of mortality [3]. Excessive production of pro-inflammatory cytokines like (TNF-α, IL-1β, and IL-6), chemokines, and other inflammatory molecules are associated with sepsis [4]. Cardiac or myocardial damage is none of the major complications of sepsis, albeit other organs like lungs, liver, and brain are affected depending on the severity of inflammation [5]. Sepsis-mediated cardiac damage is due to the overproduction of free radicals, which elicits oxidative and apoptotic damage to the heart and causes a wide range of physiological alterations in the heart [6]. Further elevated levels of pro-inflammatory cytokines are also a major reaction in the pathogenesis of sepsis-induced cardiac damage [7]. Lipopolysaccharide (LPS), a major antigenic component in the bacterial cell wall, is widely used in preclinical research to induce a septic reaction at high doses [8]. LPS-mediated myocardial injury is due to the accelerated generation of free radicals, which causes oxidative stress to cardiomyocytes and leads to the loss of myocardial membrane damage, increased accumulation of calcium, and mitochondrial damage [9]. Further, previous reports suggest that TNF-α and IL-6 are the main cytokines involved in the progression of heart failure phenotypes, encompassing myocardial dysfunction and progressive left ventricular dysfunction sepsis developed patients [10]. Reports show that LPS induction increases the production of TNF-α and IL-6 mediated through stimulation of nuclear factor (NF)-κB signalling pathway in cardiomyocytes [11]. Thus, prevention of inflammation, apoptosis, and oxidative stress may be clinically effective therapeutic target for the management of sepsis.

Natural products elicit potential effects in the prevention of a wide range of human diseases. For example, saffron biomolecules have been reported to employ different molecular mechanisms to alleviate gastric, colon, liver cancers [12,13], and, most recently, to target COVID-19 [14]. Food-derived proteins such as camel whey protein hydrolysates are shown to possess potent anticancer properties [15,16]. Other herbal extracts have recently been reported to ameliorate both fibrosis and liver cancer [17,18]. Recently, natural products elicit potential effects in the prevention of a wide range of cardiovascular diseases and disorders. Previous randomized clinical studies show that the combination of natural production effectively reduced severe sepsis [19]. It has been reported that Urtica Parviflora leafs significantly lowered plasma levels of triacylglycerides (TAGs) and low-density lipoproteins (LDLs) in rats treated with doxorubicin (DOX). Similar results have been reported with other natural extracts like Curcuma longa [20] and Flacourtia indica [21]. Treatment of saponins (extract of Panax notoginseng) has been reported to improve the left ventricular (LV) contractile function and modulate intracellular calcium [22]. Thymoquinone has been reported to attenuate cardiomyopathy in streptozotocin-treated diabetic rats [23]. Similarly, lycopene has been reported to attenuate diclofenac sodium and tulathromycin-associated cardiotoxicity in mice [24]. Dihydroxyphenylethanol (DOPET), also known as hydroxytyrosol, is the chief polyphenol component in olive oil [25]. Increasing studies have shown the efficacy of DOPET in the amelioration of cardiovascular diseases [26]. Further, DOPET showed a protective effect in the murine model of LPS induced sepsis by reducing inflammation [27]. In this backdrop, we have evaluated the efficacy of DOPET in the mitigation of LPS-induced cardiac damage.

## 2 Materials and methods

### 2.1 Chemicals

DOPET and LPS were obtained from Sigma-Aldrich, USA. The other reagents used in the study were obtained from Merck, USA.

### 2.2 Animals

Male Wistar rats weighing about 180–200 g (obtained from the Animal Center of the Shandong First Medical University, China) were used in the present study. Rats were segregated into four groups, namely, control group, LPS group, DOPECT group and DOPECT + LPS group. The animals were housed in a room maintained at a standard temperature of 23 ± 1°C and subjected to 12 h dark/light cycle. All rats were adapted to laboratory environment for 7 days.


**Ethical approval:** The research related to animal use has been complied with all the relevant national regulations and institutional policies for the care and use of animals, and were approved by the Animal Care and Use Committee of Shandong Medical University, China.

### 2.3 Study design

Control rats received saline for 7 days; the LPS group received LPS (15 mg/kg; i.p.) on 7th day; the DOPET group received DOPET (10 mg/kg; i.p.) for 7 days; the DOPET + LPS group received DOPET (10 mg/kg; i.p.) for 7 days followed by LPS (15 mg/kg; i.p.) on 7th day. After the experimental period, the animals were anesthetized, and the blood was withdrawn by retro orbital puncture, and the serum was separated after centrifugation. The animals were euthanized by cervical decapitation, and the heart was removed. Then, about 100 mg of the heart tissue was homogenized in 10% w/v cold Tris-HCl buffer and centrifuged at 4°C. After centrifugation, the supernatant was employed for biochemical analysis.

### 2.4 Assessment of cardiac injury

To assess the cardiac damage severity, the cardiac marker enzymes, such as CK-MB and LDH, were measured in serum based on the instructions provided in the kits procured from Nanjing Jiancheng Bioengineering Institute, Nanjing, China. The serum levels of cardiac Troponin T (cTnT) were analyzed based on ELISA assay as per the information provided in the kit obtained from Roche Diagnostics, Mannheim, Germany.

### 2.5 Assessment of cardiac antioxidants

The hearts were removed from the experimental rats after being sacrificed by decapitation and placed on ice, followed by washing with ice-cold saline. Next, we prepared 10% homogenate in potassium phosphate buffer (100 mM, pH 7.5). Centrifugation at 1,000×*g* for 20 min was performed at 4°C. Finally, the supernatant was collected for performing the assay. The cardiac levels of anti-oxidants like GSH, SOD, GPx, and CAT were evaluated as per the instruction provided in the kits procured from the Nanjing Jiancheng Bioengineering Institute, Nanjing, China.

### 2.6 Analysis of oxidative stress

The oxidative stress markers such as MDA and NO in the cardiac tissue were estimated according to the instructions provided in the kit procured from Nanjing Jiancheng Bioengineering Institute, Nanjing, China.

### 2.7 Serum levels of pro-inflammatory cytokines

From the experimental rats, blood was taken from the portal vein using a 1 mL syringe. It was followed by centrifugation of blood samples at 12,000 rpm for 15 min at 4°C. Collection of serum was performed in a new Eppendorf tube. The serum levels of TNF-α and IL-6 were measured by the ELISA method based on the information provided in the kit (R&D, Minneapolis, MN, USA).

### 2.8 RT-PCR analysis of inflammatory mediators

The mRNA levels of TNF-α, IL-6, NF-κB, and β-actin were analyzed by the reverse transcription (RT) PCR method. Total RNA from the cardiac tissue was extracted using the Trizol reagent as per instruction provided in the kit supplied by Invitrogen. The genes expression of the target proteins was performed by synthesizing cDNA from the isolated RNA (1 µg) using the PCR primers procured from SA Biosciences. The following PCR conditions were used in the study, denaturation temperature (95°C) for 5 min, 40 cycles, each 30 s (95°C), annealing temperature at 65°C for 30 s at 60°C of annealing temperature, and ended with an extension temperature of 72°C for 10 min. The fold changes were measured using relative band intensity.

### 2.9 Detection of apoptosis by western blotting method

The extraction of cardiac tissue proteins was done by using RIPA lysis buffer (Beyotime, China). The protein levels of BCL-2, Bax, and caspase-3 were determined by the SDS PAGE electrophoresis procedure and SDS-PAGE (10%) was employed during the estimation. The following primary antibodies were employed during the analysis: rabbit anti-Bcl-2 (1:400), rabbit anti-Bax (1:400), rabbit anti-caspase-3 (1:400), and rabbit anti-β-actin (1:400, Santa Cruz, USA). The horse radish peroxidase-conjugated goat-anti-rabbit (Santa Cruz, USA) antibody was used as a secondary antibody and incubated at room 37°C for 2 h. ECL kits were used to visualize the protein bands in the membrane and analyzed using the FR-200 system (Shanghai FURI Technology).

### 2.10 Statistical analysis

GraphPad Prism 6 (GraphPad Software, La Jolla, CA) was used for all statistical analyses. The data are expressed as mean ± standard deviation (*n* = 3) and were compared using one-way analysis of variance (ANOVA) and Bonferroni multiple comparisons test. Differences were taken as significant statistically at *p* < 0.05.

## 3 Results

### 3.1 DOPET effectively reduced the serum levels of LDH, CK-MB, and troponin-T

In this study, toxic doses of LPS significantly (*p* < 0.05) elevated the serum cardiac markers levels of LDH, CK-MB, and troponin T in the control rats. However, supplementation with DOPET significantly (*p* < 0.05) decreased the concentrations of LDH, CK-MB, and troponin T in LPS-intoxicated rats (Table 1).

**Table 1:** Effect of DOPET on cardiac markers in LPS-mediated cardiac damage

Groups	LDH (IU/L)	CK-MB (IU/L)	Troponin T (ng/mL)
Control	146.25 ± 5.43	111.76 ± 6.25	0.12 ± 0.02
LPS	564.98 ± 10.76^a^*	312.18 ± 12.76^a^*	0.76 ± 0.08^a^*
DOPET	150.85 ± 4.62	114.54 ± 7.12	0.16 ± 0.02
LPS + DOPET	256.87 ± 7.56^b^*	187.10 ± 8.45^b^*	0.35 ± 0.03^b^*

Data are depicted as mean ± SEM (*n* = 10).

^a^LPS vs control; ^b^LPS + DOPET vs LPS; *significant (*p* < 0.05). LDH, lactate dehydrogenase; CK-MB, creatine kinase-MB.

### 3.2 DOPET effectively reduced LPS-induced oxidative stress and inflammatory cytokines levels in serum

**Figure 1:**
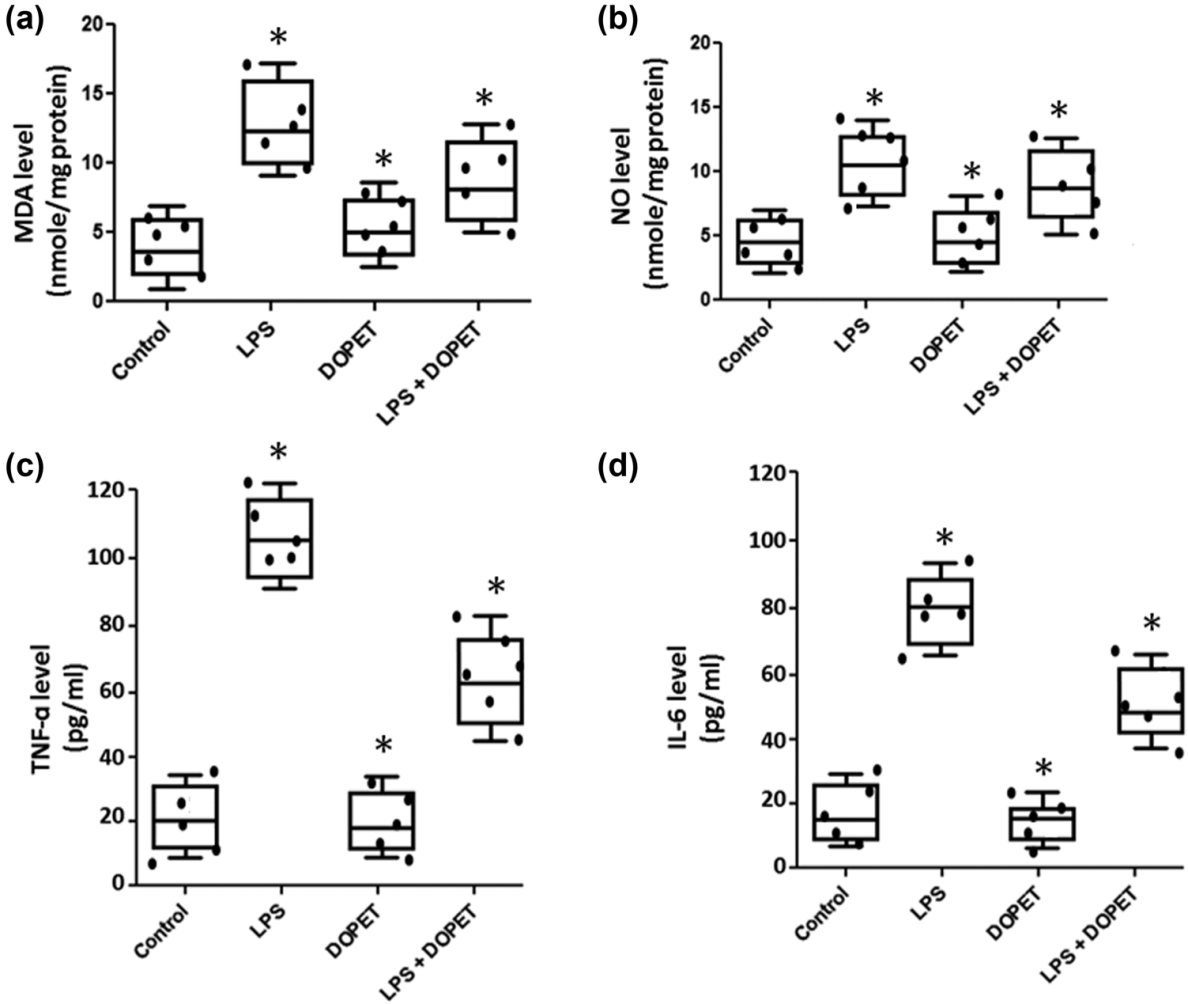
(a and b) Effect of DOPET on lipid peroxidation and nitric oxide level in cardiac tissues. Data are depicted as mean ± SEM (*n* = 10). (a) LPS vs control; (b) LPS + DOPET vs LPS. * Significant (*p* < 0.05). MDA: malondialdehyde (nmol/mg protein); NO: nitric oxide (nmol/mg protein). (c and d) Effect of DOPET on the levels of TNF-α and Il-6 in serum. Data are depicted as mean ± SEM (*n* = 10). (a) LPS vs control; (b) LPS + DOPET vs LPS. * Significant (*p* < 0.05). TNF-α: tumour necrosis-α (pg/mL); IL-6: interleukin-6 (pg/mL).

LPS intoxicated rats revealed significant (*p* < 0.05) elevated MDA levels (lipid peroxidation marker) and NO in heart homogenate as that of the control group. Of note, DOPET administration effectively decreased the MDA and NO levels to normal and thus prevented oxidative stress (Figure 1a and b). LPS significantly (*p* < 0.05) increased the serum pro-inflammatory cytokines levels such as TNF-α and IL-6 as compared to the control rats. Meanwhile, DOPET administration to LPS challenged rats significantly reduced the level of the inflammatory cytokine to normal (Figure 1c and d).

### 3.3 DOPET increased the anti-oxidant status in cardiac tissues

Toxic insult of LPS showed a significant (*p* < 0.05) reduced level of anti-oxidants SOD, CAT, GPx, and GSH in the cardiac tissue homogenate as that of the control group. Meanwhile, DOPET administration effectively (*p* < 0.05) increased the anti-oxidant level to normal as that of the LPS-intoxicated rats (Table 2).

**Table 2:** Effect of DOPET on the anti-oxidant level in LPS mediated cardiac damage

Groups	SOD (U/mg protein)	CAT (nmol H_2_O_2_/min/mg protein)	GPx (nmol CDNB/min/mg protein)	GSH (µmol GSH/mg protein)
Control	7.85 ± 0.52	6.56 ± 1.05	5.45 ± 0.72	2.75 ± 0.56
LPS	2.05 ± 0.19^a^*	3.12 ± 0.65^a^*	2.54 ± 0.24^a^*	0.65 ± 0.08^a^*
DOPET	7.19 ± 0.55	6.12 ± 0.72	5.07 ± 0.52	2.56 ± 0.32
LPS + DOPET	6.12 ± 0.42^b^*	5.86 ± 0.0.98^b^*	4.45 ± 0.36^b^*	2.12 ± 0.43^b^*

Data are depicted as mean ± SEM (*n* = 10).

^a^LPS vs control; ^b^LPS + DOPET vs LPS; *significant (*p* < 0.05). SOD: superoxide dismutase; CAT: catalase; GPx: glutathione peroxidase; GSH: reduced glutathione.

### 3.4 DOPET reduced the mRNA expression of TNF-α, IL-6, and NF-κB in LPS-challenged rat

**Figure 2:**
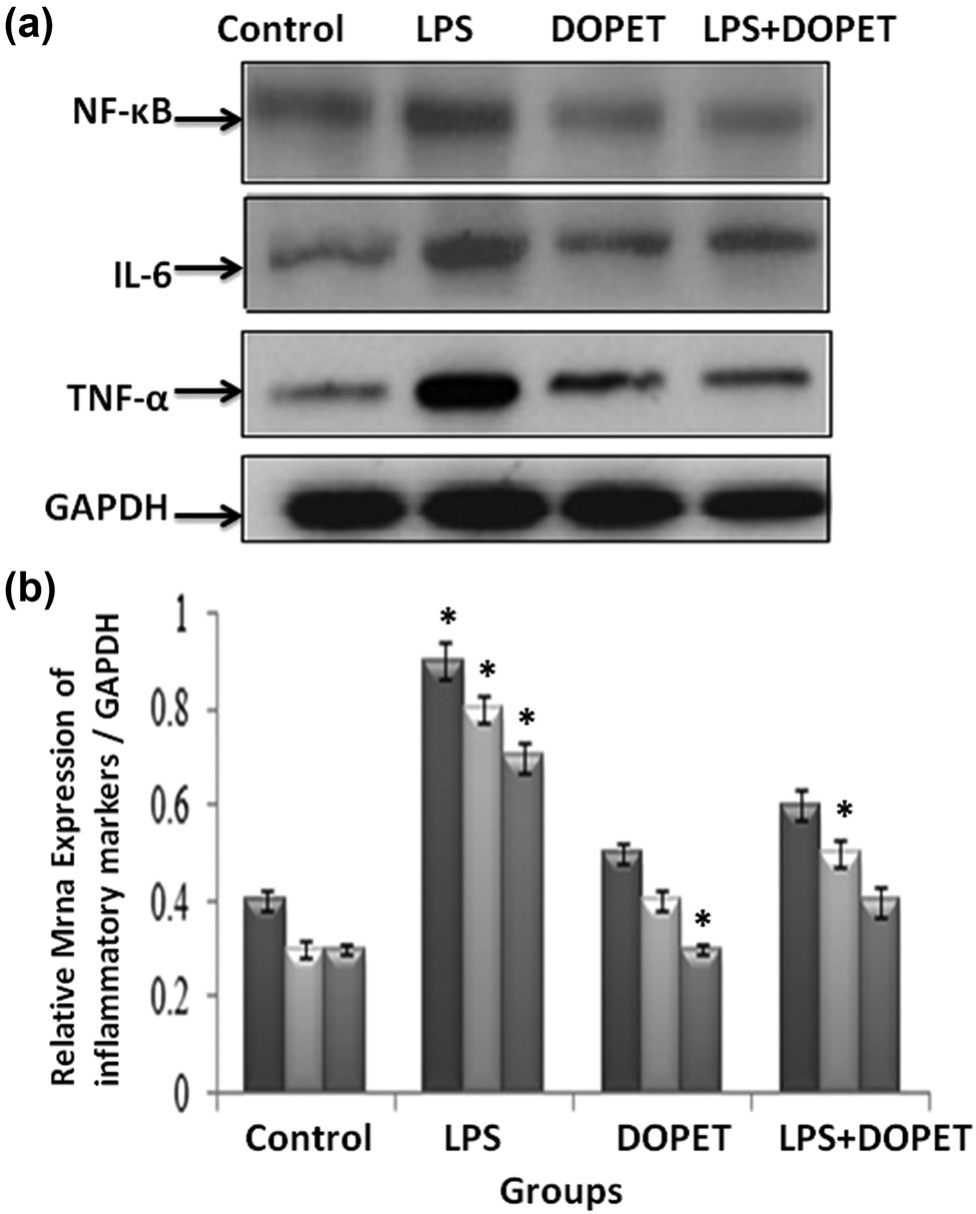
Effect of DOPET treatment on mRNA expression of inflammatory mediators. LPS-intoxicated rats showed significant (*p* < 0.05) upregulation of TNF-α, IL-6, and NF-κB, and treatment with DOPET markedly reduced the expression to normal (2a). Relative mRNA expression of inflammatory mediators (2b). Data are depicted as mean ± SEM (*n* = 3). (a) LPS vs control; (b) LPS + DOPET vs LPS. * Significant (*p* < 0.05).

The cardiac tissue mRNA levels of TNF-α, IL-6, and NF-κB were effectively (*p* < 0.05) upregulated in LPS-intoxicated rats. However, DOPET administration significantly (*p* < 0.05) downregulated the expression and thus prevented the inflammation as compared to the LPS-induced cardiotoxicity rats. The expression and relative fold change are displayed in Figures 2a and b, respectively.

### 3.5 DOPET reduced cardiac apoptosis in LPS-challenged rats

**Figure 3:**
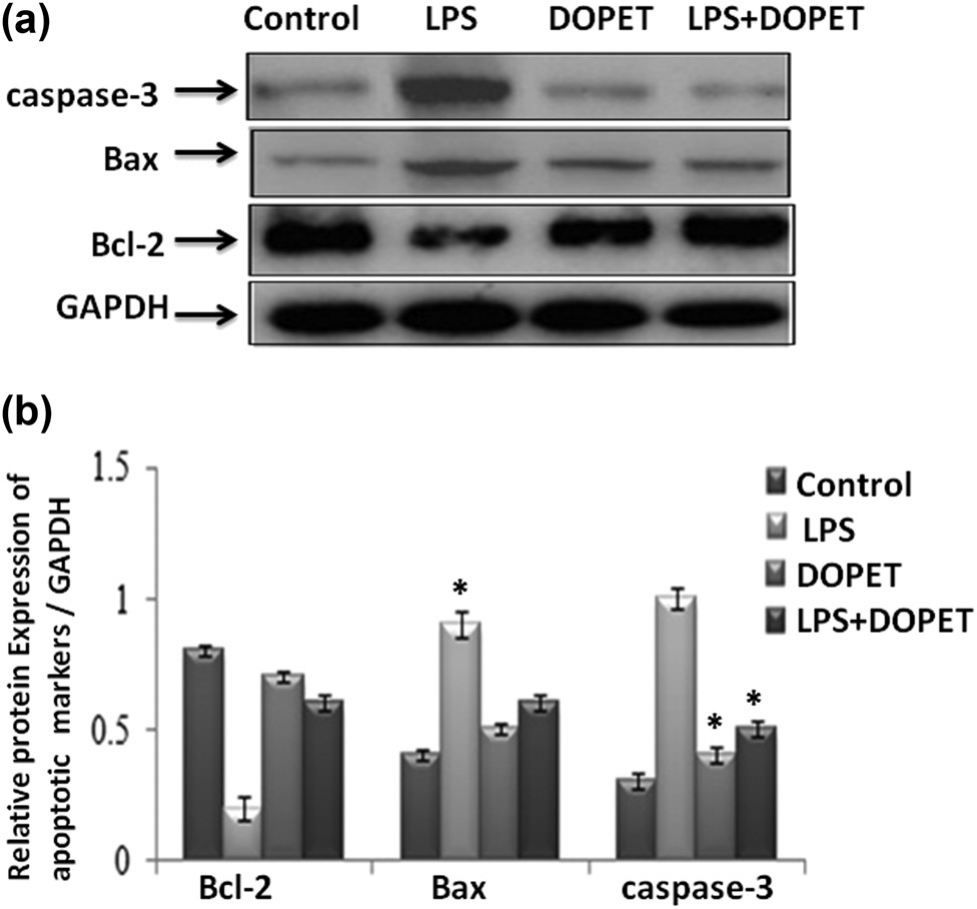
Effect of DOPET treatment on protein expression of apoptotic markers. LPS-intoxicated rats showed significant (*p* < 0.05) upregulation of Bax, caspase-3, and downregulation of Bcl-2 and treatment with DOPET markedly restored the expression to normal (a). Relative mRNA expression of apoptotic markers (b). Data are depicted as mean ± SEM (*n* = 3). (a) LPS vs control; (b) LPS + DOPET vs LPS. * Significant (*p* < 0.05).

In this study, the protein expression of apoptotic markers (Bcl-2, Bax, and caspase-3) were evaluated by the western blot method. The LPS-intoxicated rats displayed a decreased level of Bcl-2 and increased level of Bax and caspase-3, and it was significant (*p* < 0.05) as that of the control group. DOPET administration effectively (*p* < 0.05) elevated the protein level of Bcl-2 and decreased the levels of Bax and caspase-3. The protein expression of apoptotic markers and the relative fold change are shown in Figures 3a and b, respectively.

## 4 Discussion

Sepsis is an alarming clinical condition that causes multiple organ failure due to the alteration in the immune system due to infection [28]. Sepsis is one of the major reasons for global mortality, with a mortality rate of 30% [29]. Septic cardiomyopathy (SCM) is the most prevalent condition among septic patients, with an increased mortality rate between 70 and 90% [30]. LPS is a major endotoxin located in the outermost membrane of Gram-negative bacteria, which mediates the progression of many pathological conditions by increased pro-inflammatory cytokines and oxidative stress [31]. Thus, LPS is widely used in preclinical studies to study the effect of new drugs in preventing sepsis. DOPET, a potent polyphenol present in olive oil, exerts cardiovascular benefits [32]. The present study highlights that DOPET mitigated cardiac membrane damage, oxidative stress, inflammation, and apoptosis in a murine model of LPS-induced septic cardiomyopathy.

Patients with septic cardiomyopathy showed elevated levels of cardiac markers in serum, indicating a severe loss in cardiac membrane integrity. A previous study shows the increased concentration of cTnI in septic patients without evidence of coronary heart syndrome [33]. Further, LDH and creatine kinase are the important cardiac enzymes that orchestrate ATP production and maintain cardiac membrane integrity [34]. In our study, LPS insulted elevation of serum levels of LDH, CK-MB, and cnTn1 rats, which substantiated the marked cardiac membrane damage. Treatment with DOPET markedly reduced the elevated level of cardiac markers through its membrane-stabilizing potential, concordance with the previous report [35].

LPS generated during sepsis can induce free radical production by mitochondria [36]. The released free radicals cause an imbalance between oxidants and anti-oxidants and thus affect the cardiac defense system. Lipid peroxidation is one of the major toxic events during septic cardiomyopathy since the heart contains more lipids and reduced anti-oxidant defense. Previous studies have shown the marked involvement of lipid peroxidation during sepsis conditions, and it has a significant association with mortality [37]. In our study, LPS intoxicated rats showed an increased level of MDA (lipid peroxidation markers) in the cardiac tissue reflecting the oxidative stress. Treatment with DOPET markedly reduced the MDA level to normal and thus inhibited the free radicals. The free radical inhibitory potential of DOPET is due to the presence of o-dihydroxyphenyl group, which terminates the lipid peroxidation chain by donating a hydrogen atom to a peroxyl reactive ion [38]. Further, the lipid peroxidation in turn suppresses the anti-oxidants network in the heart, which is due to the overutilization of these anti-oxidants in scavenging the free radicals generated by sepsis conditions [39]. In our study, septic cardiotoxicity rats elicited a reduced level of anti-oxidants SOD, CAT, GPx, and GSH, and DOPET treatment significantly boosted the anti-oxidants and thus reduced the role of these anti-oxidants in free radicals elimination during LPS-induced cardiac sepsis. A previous study shows that DOPET increased the anti-oxidant level in cadmium-induced cardiac oxidative stress [40].

Rampant inflammation orchestrates a major role in the progression of cardiac injury. LPS is employed to provoke inflammation response, which is evident by the increased level of pro-inflammatory cytokines [41]. Previous reports show that TNF-α is the major cytokine involved in LPS-mediated septic shock with an increased level of TNF-α in cardiac tissues and serum [42]. Further, it has been shown that LPS-induced sepsis substantially upregulates the expression of IL-6 in cardiac tissues through increased production of collagen and causes fibrosis of the heart. We found that LPS-induced sepsis elicited increased the serum level and mRNA expression of TNF-α and IL-6 in cardiac tissues. Further, DOPET administration significantly reduced the serum concentration and downregulated the mRNA expression of inflammatory cytokines. A previous study showed that DOPET reduced the protein expression of TNF-α and IL-6 in cadmium-induced cardiac oxidative stress [40].

Mounting evidence shows that NF-kB is linked to rampant stimulation of various inflammatory mediators such as cytokines and chemokines [43]. Reports indicate that blocking of NF-kB signaling leads to the reduced expression of TNF-α and IL-6 [44]. Earlier studies showed that NF-kB and MAPK signalling pathway orchestrate a pivotal role in LPS-mediated stimulation of inflammatory mediators and their transcription, which is the cardinal factor in the progression of septic cardiomyopathy [45]. The current study shows that LPS-intoxicated rats showed upregulated mRNA levels of NF-kB in cardiac tissues, and DOPET treatment inhibited the upregulation of NF-kB and thus prevented the inflammation in septic cardiotoxicity.

During LPS-mediated sepsis, the rampant release of inflammatory mediators imparts oxidative cardiotoxicity, further promotes cardiac apoptosis, and finally, leads to cardiac failure [46]. The Bcl-2-related apoptosis family protein encompasses anti-apoptotic protein (Bcl-2), and pro-apoptotic proteins (Bax) are the prime apoptosis regulators that orchestrate the mitochondrial apoptotic pathway [47]. The caspase protein mediates an important role in apoptosis during stress conditions. During mitochondria-mediated intrinsic apoptosis, the activation of caspase-9 occurs, which in turn activates the final effector molecule caspase-3 [48]. In this study, LPS-mediated cardiotoxicity reveals downregulated and upregulated protein expression of Bcl-2 and Bax, and caspase-3, respectively. Meanwhile, DOPET mitigated apoptosis by inhibiting the protein level of Bax and caspase-3 and increasing the Bcl-2 protein level. A previous study shows that DOPET reduced apoptosis in cadmium-induced cardiac oxidative stress [40].

In conclusion, DOPET mitigated LPS-mediated septic cardiotoxicity by restoring cardiac membrane integrity, preventing oxidative stress, inflammation, and apoptosis. Further studies are warranted to evaluate the involvement of various signalling pathways in septic cardiomyopathy and the effect of DOPET.
